# Improving asthma outcomes: Clinicians’ perspectives on peripheral airways

**DOI:** 10.1016/j.jacig.2024.100228

**Published:** 2024-02-13

**Authors:** Gregory G. King, Li Ping Chung, Omar S. Usmani, Kris Nilsen, Bruce R. Thompson

**Affiliations:** a Royal North Shore Hospital, Woolcock Institute of Medical Research and Faculty of Health and Medicine, University of Sydney, Sydney, Australia; b Department of Respiratory Medicine, Fiona Stanley Hospital, Perth, Australia; c National Heart and Lung Institute, Imperial College London, Royal Brompton Hospital, Airways Disease Section, London, United Kingdom; d Department of Health and Medical Sciences, Swinburne University of Technology, Melbourne, Australia; e Melbourne School of Health Science, University of Melbourne, Melbourne, Australia

**Keywords:** Asthma management, small airways dysfunction, peripheral airways, treatable trait, oscillometry, multiple breath nitrogen washout, respiratory function assessment, inhaler particle size

## Abstract

Disease of the peripheral (or small) airways is fundamental in asthma, being closely related to symptoms (or lack of control of them), airway hyperresponsiveness, spirometric abnormalities, risk of loss of control, or exacerbations and inflammation. Current technology now allows routine measurement of peripheral airway function. Having a working concept of peripheral airways disease in asthma is arguably very useful to clinicians and beneficial to patients because it allows a more comprehensive assessment of asthma severity (rather than just symptoms alone, which is the norm), tracking of progress or deterioration, and assessing response to treatment. Oscillometry is a sensitive way to monitor the peripheral airways, whereas multiple breath nitrogen washout parameters are excellent measures of future risk. In the longer term, physiologic measurements will be crucial in research to define causes and find new disease-modifying treatments.

Asthma is a common and well-known diagnosis to specialists, general practitioners, and the general public.^1^ Decades of research has shed much light on the nature of asthma, but as is often the case, it raises more questions, and importantly causality and pathophysiology remain very poorly understood. Perhaps the push to simplify the concept and hence its management has been detrimental, given that its clinical spectrum is broad and complex and a simple approach to treatment does not match the reality of this complexity. The authors strongly argue that better characterization of pathophysiology, be they called phenotypes, traits, or biomarkers, is required to improve management and to advance research. Respiratory physiology has a fundamental role in diagnosis and management of airways diseases and is the functional representation of both inflammation and lung and airway remodeling. Peripheral airways disease is an integral part of asthma, and the ability to measure function of the peripheral airways has allowed studies that reveal the importance of small airway function in asthma. Is this useful for clinicians and researchers? In this article, we explain what aspects of peripheral airway dysfunction are clinically important, and how this informs assessment and management.

## The current paradigm of asthma needs to change

Asthma and airways diseases in general are complex and multifaceted. Asthma is characterized by variable airway narrowing, structural remodeling, inflammation that is heterogeneous between individuals, airway hyperresponsiveness, and, in some, excessive loss of FEV_1_. Asthma guidelines for clinical assessment and management have been around for decades and have gone through many iterations. There are international guidelines as well as local modifications of the international guidelines, which have led to a great focus on managing symptoms in day-to-day general practice. The Global INitiative for Asthma and the Asthma Management Handbook define asthma by symptoms and supportive tests, which in practice means that asthma is diagnosed by history and examination before embarking on treatment; objective tests are rarely used in general practice.^2^ Asthma severity is currently determined by prescribed medications (high-dose inhaled corticosteroid [ICS]/long-acting β_2_-agonist [LABA] indicates severe asthma), without use of objective tests. The major flaw is that many clinicians experienced in managing asthma know that symptoms are often very deceiving. Poor perceivers of bronchoconstriction are undertreated, whereas others with little or no asthma may be overtreated for symptoms. The lack of objective measurements in clinical practice is a major problem causing underdiagnosis and overdiagnosis and suboptimal treatment.

Current physiologic tests are very useful and should be more frequently used, but this requires identification of barriers to use and better education to allow clinicians to apply them. Variable airway narrowing can be assessed by bronchodilator responsiveness (BDR), but this is insensitive and nonspecific. Bronchial challenge testing is poorly understood by clinicians and, like any test, must be interpreted in its clinical context and is not a diagnostic test in itself. Peak flow monitoring is not commonly used; it is somewhat difficult for some patients given its maximal forced expiratory maneuver and is difficult for general practitioners to interpret. Fortunately, sophisticated daily electronic lung function monitoring techniques are on the horizon that will dramatically improve the way in which we characterize and manage asthma.

## The future: Comprehensive phenotyping including peripheral airways

The relatively recent concept of treatable traits has gained increasing interest, and its relevance and importance are recognized in the Global INitiative for Asthma and local guidelines. Clinicians are increasingly recognizing the applicability and usefulness of the concept in routine practice. Identifying what is treatable improves important patient outcomes, for example, obesity, deconditioning, and rhinitis. Peripheral airway function should be part of the treatable traits approach to management and is arguably a specific treatment target. It could be thought of in the same way as variable airway narrowing, airway hyperresponsiveness, and FEV_1_ impairment. It is also critical in the development of future treatments—either inhaled or systemic. Like any asthma trait, it varies between individuals and has clinical correlates.

One of our many challenges is to work out how to make “untreatable traits” treatable (eg, fixed airflow obstruction, type 2 low disease, and mucus hypersecretion). Fixed airflow obstruction occurs in up to a third of people with asthma, even in those labeled as “mild asthma.”^3^ It can be debilitating and is complex in pathophysiology in which peripheral airway dysfunction is fundamental.^3^, ^4^, ^5^ Although long-acting muscarinic antagonists are particularly beneficial when there is fixed airflow obstruction,^6^^,^^7^ suggesting parallels with smoking-related obstructive disease, treatments that are disease-modifying or curative remain greatly needed. Arguably, the key to finding treatments and managing this major problem will be in the peripheral airways. Peripheral airway measurements could and should be part of routine practice in the near future.

### What are peripheral or small airways?

Small airways can be defined in different ways, but precise anatomical definitions are probably not necessary. Because we can “see” subsegmental airways bronchoscopically down to about generation 4, and by computed tomography (CT) down to generation 6 or so, the concept that these are proximal airways or “large” airways seems an intuitive and clinically pragmatic definition. We can diagnose bronchiectasis, dynamic airway collapse, and mucus plugging in these airways that are easily “seen.” Beyond these airways and into the lung periphery, functional and anatomical assessment is more complex. Structural information from high-resolution CT is limited, and they are inaccessible to current bronchoscopic imaging although advanced techniques in development, such as polarization-sensitive optical coherence tomography,^8^ might change this. Spirometry is insensitive to peripheral airway function, whereas tests such as respiratory oscillometry and multiple breath nitrogen washout (MBNW) have proven to be clinically informative, even though they are not specifically or unequivocally measures of peripheral airway function. For this article, we refer to peripheral airways as defined earlier, rather than to small airways, although for most intents and purposes, the labels are interchangeable.

### Clinical relevance of peripheral airways in asthma

An understanding of the role of peripheral airways disease in asthma is important in clinical practice and research. Knowledge of peripheral airway dysfunction allows clinicians to have a comprehensive understanding of asthma because it is fundamental to (1) pathophysiology (bronchoconstriction and airway hyperresponsiveness), (2) defining clinical phenotypes (particularly when taking a “label-free” approach), and (3) disease severity and rational approaches to drug treatment. Asthma involves the entire airway tree, a finding that is consistent between CT imaging,^9^ biopsy,^10^ and postmortem studies^11^ and applies even in mild asthma.^12^^,^^13^ The contribution of peripheral airway dysfunction however varies, which influences clinical disease, that is, symptoms, severity, exacerbation risk, response to treatment, and lung function loss.

Peripheral airway dysfunction plays a critical role in severe or treatment-resistant asthma,^12^, ^13^, ^14^ wherein there is persisting airway hyperresponsiveness, episodic bronchoconstriction, symptoms, exacerbations, and loss of lung function^13^ despite optimal inhaled treatment. Severe inflammation and remodeling in peripheral airways, for example, when exhaled nitric oxide levels are high and peripheral airway function tests indicate severe impairment, will be difficult to target by ICSs/LABAs. Poor gas mixing means that the worst affected airways have the poorest ventilation, making aerosol targeting difficult. Systemic treatment may be effective because of its ability to access the peripheral airway compartment (ie, oral corticosteroids, macrolides, and biologics).^15^ Targeting peripheral airways, particularly those regions where ventilation is poor, should inform treatment in the future, that is, a precision medicine approach. Targeting peripheral airways is discussed later herein.

Although complete control of certain features of disease can be achieved in asthma, true disease modification (eg, prevention of joint deformity in rheumatoid arthritis) is not achievable with current treatment.^16^ In most patients, lung function normalizes and symptoms and exacerbations are abolished with continuous treatment, and whether this constitutes disease remission is semantic. Cessation of treatment results in a return to the pretreatment state, and ICS treatment only partially improves airway remodeling.^17^ Long-acting bronchodilators improve but do not normalize lung function in asthmatic patients who have persistent airflow limitation.^18^ Arguably, remission or cure requires treatments that specifically address remodeling and reverse or prevent tissue repair and proliferation in peripheral airways. A major barrier to developing specific treatments for remodeling is its complexity, our poor understanding of mechanisms, and our inability to measure it longitudinally.

An important clinical consequence of remodeling is lung function loss. Impaired FEV_1_ involves the entire airway tree, including peripheral conducting and gas-exchanging (acinar) airways and the lung parenchyma, which is evident by the loss of lung elastic recoil and lung density.^4^^,^^11^^,^^19^, ^20^, ^21^ Furthermore, the paradigm that remodeling is caused by type 2 inflammation, implying that controlling type 2 inflammation reverses or halts remodeling, is flawed. Type 2 inflammation is common in people without asthma, whereas type 2 low inflammation is also common in those with asthma. To address remodeling in asthma (hence inducing disease remission and prevent or reverse FEV_1_ loss) requires an ability to probe the function of the entire airway tree and lung parenchyma and relate this to cellular processes responsible for tissue repair, proliferation, and inflammation.

### Measurement of peripheral airway function

Both respiratory oscillometry and MBNW are quite different to spirometry, being done during relaxed, near-tidal breathing, whereas spirometry is a forced, maximal maneuver. Both tests are sensitive to an increase in the *heterogeneity* of airway caliber and elasticity of the lungs. Pragmatically, both tests are sensitive to the heterogeneity (or degree of variation) in airway caliber, particularly of the more peripheral airways as well as the lung tissues. Heterogeneity is a core feature of airways disease, and hence oscillometry and MBNW are physiologically relevant measures of peripheral airway function. For interested readers, we provide the description and principles of 3 main methods of peripheral airway measurement: MBNW, respiratory oscillometry, and ventilation imaging.

#### Multiple-breath nitrogen washout

The MBNW is a measure of impaired gas mixing being a functional consequence of heterogeneous ventilation caused by peripheral airways disease. The MBNW is performed by “washing out” the alveolar nitrogen by continuous breathing of pure oxygen. The original method used a single vital capacity breath of pure oxygen (single-breath nitrogen washout) and was modernized to the MBNW through computational models of ventilation distribution.^22^ The MBNW entails repeated, near-tidal volume breath that progressively washes the alveolar nitrogen out of the lung. The shape of the nitrogen versus exhaled volume curve of each breath (alveolar phase III slope) is analyzed on the basis of the principle that more heterogeneous ventilation due to peripheral airways disease causes the nitrogen concentration to rise more during exhalation (steeper phase III slope). It also takes more breaths to wash out all of the nitrogen (less efficient gas mixing).

The phase III slope analyses provide indices of impaired gas mixing in peripheral airways that are compartmentalized into small conducting airways (Scond), that is, non–gas-exchanging, and more peripheral acinar (gas-exchanging units) zone of the lung (Sacin). The lung clearance index is a global measure, akin to the combined effects of Scond and Sacin on overall ventilation. It indicates how much ventilation is required to completely wash out all the nitrogen from the lungs, normalized to lung size (as measured by functional residual capacity). For example, if 16 L of pure oxygen is breathed to wash out a functional residual capacity of 4 L, then the lung clearance index is 16 ÷ 4 = 4 “lung turnovers.” The units of Scond and Sacin are somewhat unintuitive (L^−1^) and small in value, being about 0.03 L^−1^ for Scond and 0.13 L^−1^ for Sacin. Perhaps changing units to larger values (eg, 3 L^−1^ and 13 L^−1^) might make it less obscure. Higher numbers essentially represent steeper phase III slopes, more impaired gas mixing, and worse heterogeneity. Therefore, worse peripheral airway function means that nitrogen concentration (measured as a percentage) rises more over the course of exhalation (measured in liters), and hence the unit is “per liter of exhaled breath.”

**Fig 1:**
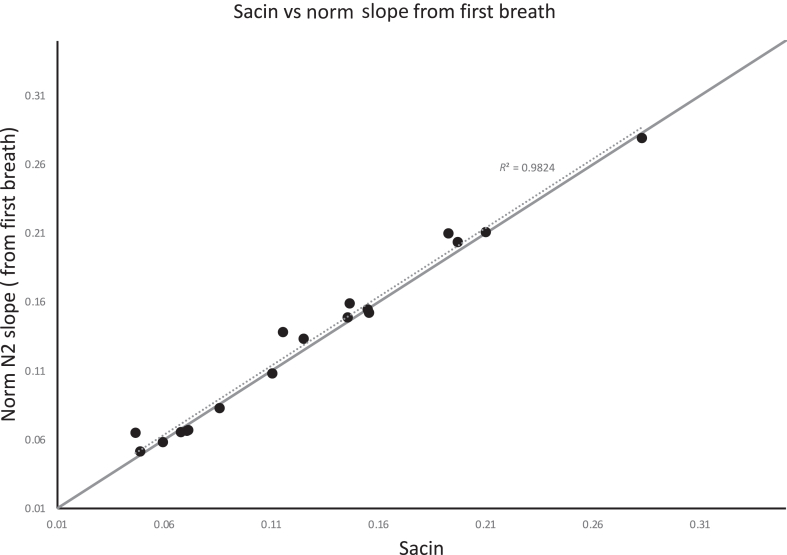
The relationships between the normalized slope from the first washout breath compared with Sacin calculated from the full washout to expired nitrogen concentration of 1/40th of starting concentration. Each subject completed 3 washouts, and the mean of the tests is displayed.

Utility of any test requires it to be practical in everyday use, and it is probably necessary to shorten the test to make it practical in routine lung function testing laboratories. Greater ventilatory impairment means more breaths are needed to get to the test end point: 1/40th of the starting nitrogen concentration. Also, longer reequilibration times are then needed between replicate washouts. A washout of only several breaths may be enough to calculate the MBNW parameters and requires development. An example of potential feasibility is demonstrated in Fig 1. As a pilot we reanalyzed MBNW from 18 subjects with asthma and used only 1 breath to estimate Sacin from the normalized nitrogen slope. Although there is a small offset that is likely to be immaterial, the correlation is very strong. These results are encouraging and warrant further exploration to enable a shorter efficient MBNW test.

#### Respiratory oscillometry

Oscillometry is performed by imposing a pressure oscillation onto normal breathing. This produces a corresponding flow and volume change. It is fast and easy to administer, for example, 3 to 4 acquisitions of 20 to 30 seconds each or a single 1-minute recording. However, as with any lung function test, technical skill is essential to obtain high-quality recordings, because abnormal breathing patterns, swallowing, and incorrect tongue position lead to artifacts and inaccurate measurements.^23^, ^24^, ^25^

Most oscillometry devices use sinusoidal oscillation, whereas the impulse-oscillometry system uses discrete 5-Hz impulses. Both techniques are considered oscillometry, irrespective of the oscillation waveform because the fundamental concepts are identical wherein complex calculations allow derivation of the core oscillometry parameters from pressure, flow, and volume changes—being respiratory system resistance (Rrs; primarily related to flow change) and reactance (Xrs; primarily related to volume change). The lowest frequency used in clinical practice is 5 Hz, and Rrs and Xrs at this frequency will reflect function of most of the tracheobronchial tree, including the peripheral airways. At this frequency, higher Rrs indicates smaller overall airway caliber and more heterogeneity of caliber. Xrs is negative at 5 Hz, and lower values (more negative) indicate lower *dynamic* compliance (ie, the respiratory system is stiffer to oscillate) because of greater overall peripheral airway narrowing, with some peripheral and central airways being severely narrowed or closed (sometimes referred to as derecruitment). Importantly, Xrs does not represent *static* compliance from classic lung mechanics, which is measured by esophageal manometry during breath hold and indicates the elastic properties of the alveoli. Dynamic compliance and reactance are influenced predominantly by airways during breathing.^26^^,^^27^

**Fig 2:**
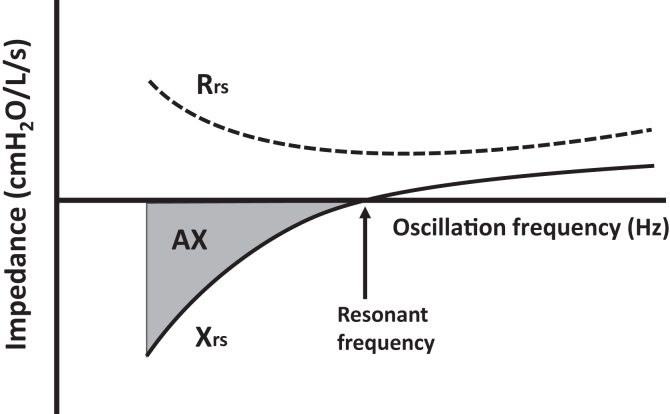
Generalized diagram of the relationship between Rrs and Xrs and frequency.

Rrs and Xrs differ, depending on the oscillation frequency; that is, they are frequency-dependent (see Fig 2). The difference between Rrs measurements at 5 Hz and 19 or 20 Hz (putatively large airway resistance) has been almost universally accepted in the literature as a specific measure of peripheral airway function. Results of computational modeling^28^ and frequency dependence both relating to forced expiratory flow at 25% to 75% of forced vital capacity^29^ somewhat support this concept. Thus, the Rrs_5_ − Rrs_19_ (or Rrs_5_ − Rrs_20_) difference probably does represent abnormal peripheral airway function in many clinical situations, but it is not as specific as many authors might suggest. Frequency dependence of Xrs, however, is handled somewhat differently to Rrs. This arose because the resonant frequency of the respiratory system (defined as the frequency at which Xrs is 0) provides an area under the curve measurement (see Fig 2) and has been named area under the reactance versus frequency curve (AX).^30^ As an integral of Xrs versus frequency, it reflects both the rightward shift of the Xrs curve (ie, increase in resonant frequency) and the decrease in Xrs at lower frequencies. Thus, the 5-Hz Xrs measurement^31^ and AX are good measures of peripheral airway function.

**Fig 3:**
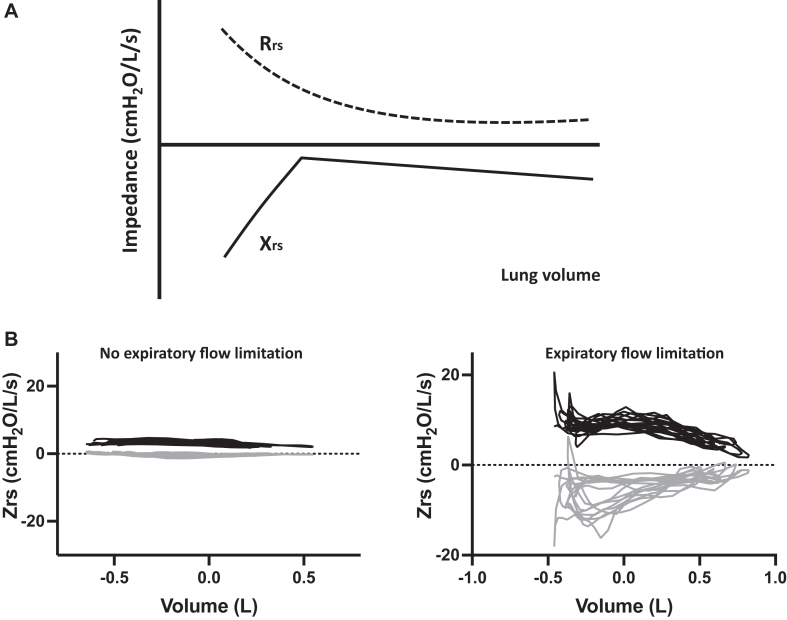
**A,** Relationships between Rrs and lung volume as well as Xrs and lung volume (volume dependence). There is an exponential relationship between Rrs and lung volume, whereas Xrs has a linear relationship, which changes dramatically at the point of lung derecruitment (airway closure). **B,** Volume dependence of Rrs and Xrs during tidal breathing. Rrs is *black* and Xrs is *gray.Zrs*, Respiratory system impedance. The left graph shows minimal change in Xrs over the breathing cycle, indicating no expiratory flow limitation. The right graph indicates a marked decrease in Xrs during expiration, indicating the presence of expiratory flow limitation (small airway narrowing/collapse).

Oscillometry can be reliably self-administered, and thus it is suitable for home monitoring of lung function.^32^, ^33^, ^34^ It may be the best tool for measuring day-to-day changes in airway function; affordable, hand-held devices are imminent. In addition, because Rrs and Xrs measurements have high temporal resolution (about 5 measurements per second), changes during breathing “within-breath” analyses^35^ and volume dependence^36^, ^37^, ^38^ can be measured (see Fig 3). These parameters allow further characterization of peripheral airway function that is clinically relevant in relation to asthma severity and control.^34^

#### Ventilation imaging

**Fig 4:**
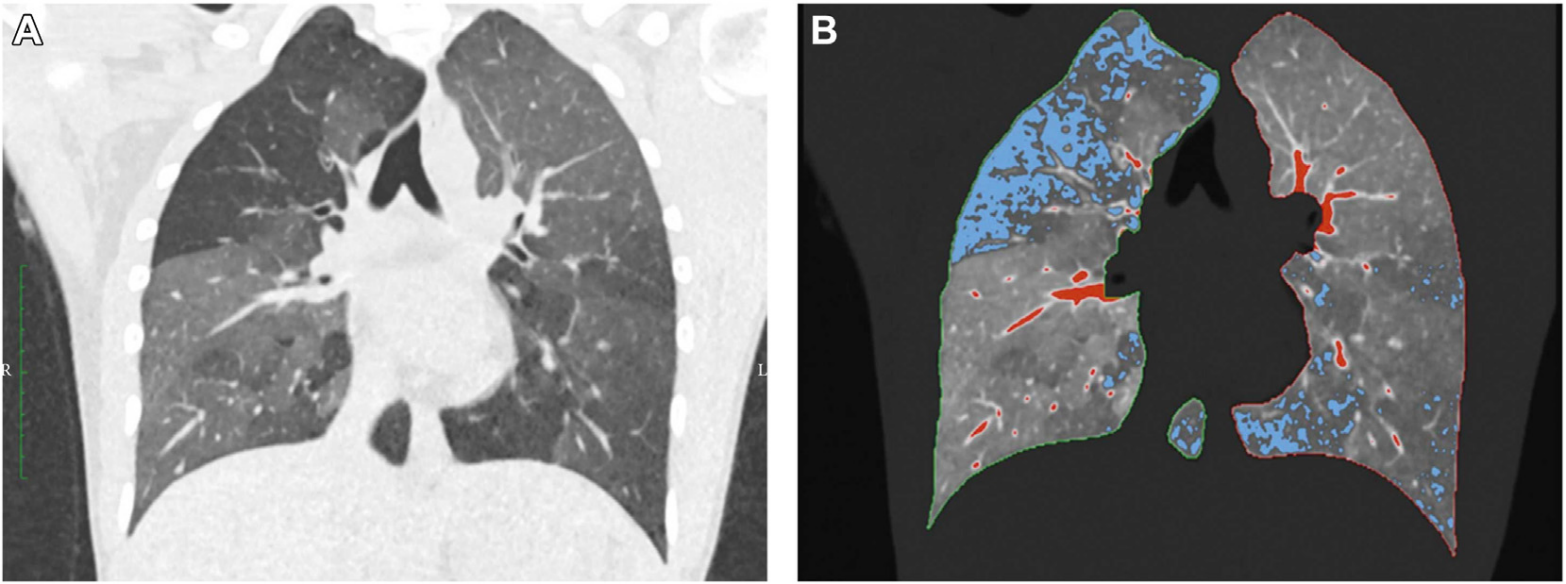
**A,** Expiratory CT scan showing areas of gas trapping due to severe narrowing of small airways during expiration. **B,***Blue* indicates areas of emphysema based on Hounsfield unit values less than −950, and *red* indicates pulmonary blood vessels based on Hounsfield unit values greater than −200. Reproduced with permission from Gawlitza et al.^40^

Images of ventilation from 3-dimensional functional imaging likely reflect small airway function.^39^ Heterogeneity of ventilation can be captured in 3-dimensional images using a marker to track air movement, such as a radionuclide in nuclear imaging (single-photon emission computed tomography [SPECT] or positron emission tomography [PET]), by hyperpolarized gases or pure oxygen in magnetic resonance imaging (MRI) and by changes in lung density using dynamic CT (see Fig 4). Most of the published studies look at nonventilation (ie, airway closure or “ventilation defects”), whereas the functional and clinical significance of reduced ventilation has been little studied.

**Fig 5:**
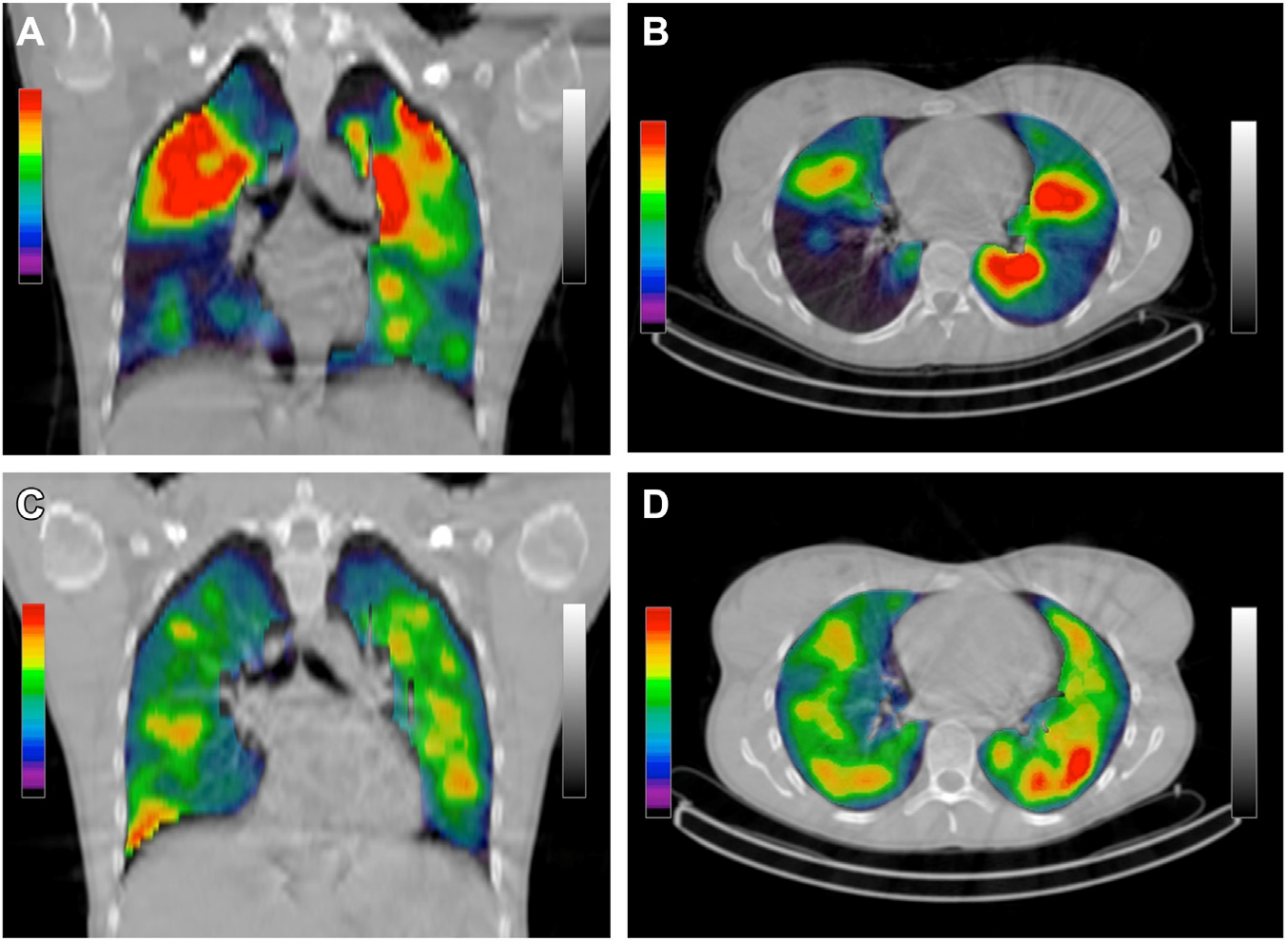
Technegas ventilation SPECT image showing the distribution of ventilation in upright posture, before and after bronchodilator inhalation. Female subject aged 28 years with asthma. **A** and **B**, Baseline images during bronchoconstriction (FEV_1_ of −1.99 *z* scores). **C** and **D**, Postbronchodilator images after improvement in FEV_1_ of 28% of baseline value.

SPECT ventilation imaging is best done using Technegas (Cyclomedica Australia, Kingsgrove, Australia), which is an ultrafine, submicron carbon particle containing 99mTc,^41^ which allows upright inhalation. Once inhaled, the particles remain stationary so that although scanning is in supine position, the radioactive distribution represents upright inhalation. Fig 5 shows a TechnegasSPECT imaging of a patient with asthma, before and after bronchodilator inhalation. All other imaging techniques measure supine ventilation because gases are inhaled at the time of scanning. Because Technegas is used routinely across many countries for diagnosis of pulmonary embolism, it has the potential for routine use in airways disease. PET is another nuclear imaging modality that could be used for ventilation imaging. Gallium-68 has been incorporated into the same particles that comprise Technegas (“Galligas”) for inhalation and makes use of the greater spatial resolution of the PET camera. PET camera and Gallium-68 are, however, less available than SPECT with Technegas using 99mTc.

Similarly paired inspiratory and expiratory CT could be used to provide quantitative indices of gas trapping if the required software for the complex analyses can be implemented commercially. Expiratory gas trapping in asthma reflects predominantly severe narrowing and closure of peripheral airways, and gas-trapping indices correlate with exacerbation and hospitalization risk, forced expiratory flow at 25% to 75% of forced vital capacity, oscillometry parameters, residual volume/total lung capacity, airway hyperresponsiveness,^9^^,^^42^, ^43^, ^44^ and phase III slope of the single-breath nitrogen washout.^45^^,^^46^

MRI is costly and technically difficult and complex to conduct, particularly in the administration of the imaging agents. Helium 3 (He3) is inaccessible for routine clinical use; 129Xe is more accessible but hyperpolarization is thus far not freely available nor practical. Pure oxygen is a possible alternative, but there are also challenges to administering it in an MR scanner and in the complex data processing. Indices of regional ventilation from MRI studies using pure oxygen correlate well with indices derived from MBNW,^47^ whereas poorly ventilated regions measured in hyperpolarized He3 MRI correlate with oscillatory reactance.^48^^,^^49^ The heterogeneity of ventilation measured by coefficient of variation in He3 MRI (which reflects peripheral airways disease) also relates to oscillometry parameters and to airway hyperresponsiveness.^50^

Therefore, MBNW, oscillometry, and functional imaging show strong relationships with clinically important indices, even stronger than for spirometry. The application of these outcomes to develop new drugs and treatments seems obvious because they would greatly improve on our current standard of symptoms, exacerbations, and FEV_1_, which are clearly inadequate. This remains our biggest challenge and impediment to progress.

### Peripheral airways disease informing asthma management

Lung function measurement is of central importance in asthma management and should always be interpreted within the clinical context,^51^ for example, lung function loss, exacerbation history, current inhaler usage, and adherence. How individual physicians act on that knowledge depends on their expertise and understanding, but patients want to know about their lung function and future risk, regardless of their doctor’s interest or otherwise. For example, coronary artery calcium scores or HbA_1c_ are routinely calculated to inform risk and to inform the need for closer monitoring or not. The same applies to peripheral airway function measurements in response to treatment and progress over time. A physician may or may not increase treatment or change an asthma action plan on the basis of a decrease in oscillatory impedance or observation of a large bronchodilator response, depending on the clinical picture.

Spirometry will remain our criterion standard for the foreseeable future, but peripheral airway measurements complement spirometry, and together they provide a comprehensive picture of function. We hope that these are reasons for clinicians to further explore and use peripheral airway function tests to inform management. The following are suggestions on how to use peripheral airway tests to inform clinical decisions.

#### Multiple-breath nitrogen washout

Small airway function measured by MBNW is clinically useful because of its associations with airway hyperresponsiveness,^52^^,^^53^ asthma control,^54^^,^^55^ and risks of exacerbation.^55^^,^^56^ Worse peripheral airway function measured by MBNW indicates incomplete response to current ICS treatment and is predictive of worsening asthma when ICSs are reduced.^57^ Furthermore, MBNW parameters improve with treatment in parallel with improved symptoms and spirometry.^54^^,^^58^^,^^59^ Scond and Sacin may decrease (ie, improve) by 1.7 and 0.7 *z* scores, respectively, with ICS/LABA treatment in subjects with moderate asthma.^59^ Scond appears to relate to symptom control, because improvement with treatment (decreased value) is associated with improved symptom control. Sacin, however, appears to relate to exacerbation risk, with high values being associated with exacerbations in the past year^56^ and loss of control with ICS downtitration.^57^

Sacin greater than 6.84 *z* scores has an 89% specificity and 56% sensitivity in separating people with asthma who have not exacerbated from those who have in the previous year.^56^ It is uncertain whether improvements in spirometry or MBNW indices (or oscillometry parameters) indicate reduced exacerbation risk. Nevertheless, it is likely that improved MBNW parameters, particularly of Sacin and especially if it normalizes, indicate excellent responses to treatment and reduced risk of exacerbation.^56^^,^^57^ For example, Sacin greater than 6.84 *z* scores, which fails to improve with treatment as mentioned earlier, would indicate the need for increased treatment, closer monitoring, and an effective asthma action plan.

#### Respiratory oscillometry

Oscillometric parameters are abnormal in a similar proportion of subjects with asthma as spirometry, but there is considerable discordance.^60^ There are now several published predictive equations to determine limits of normality.^23^^,^^61^, ^62^, ^63^, ^64^ We looked at data from our recent publication of spirometry and oscillometry in a community sample of more than 1000 adults 40 years and older, which included 122 individuals with asthma.^63^ The prevalence of reduced lung function, as determined by *z* scores for FEV_1_ and FEV_1_/forced vital capacity ratio, were 20% and 26%, respectively. The prevalence of abnormal Rrs and Xrs was similar at 18% and 28%, respectively. However, only about a third had concordant results, which indicates that Rrs and Xrs represent different physiologic function compared with spirometry. A comparison of abnormal Rrs_5_ − Rrs_20_ to 3 indices of midexpiratory flow from spirometry, in a random Chinese community sample 40 years and older, showed again poor concordance with a κ of 0.32.^60^ Abnormal peripheral airway function identified by midexpiratory flow indices was about 50% more prevalent than using Rrs_5_ − Rrs_20_.

In the severe asthma population, even relatively mild impairment in Xrs suggests increased exacerbation risk. Impairment of Xrs to less than −1.59 *z* scores had 61% specificity and 89% sensitivity in separating patients with asthma who had an exacerbation in the previous year from those who had not.^56^

A minimum *physiologically* important change can be determined from repeatability in healthy subjects.^58^^,^^61^ The coefficient of repeatability (wherein 95% of repeated observations lie if zero bias) for a testing session measured in 368 healthy adults is about ±1 cmH_2_O/L/s (17%) for Rrs_5_, about ±0.5 cmH_2_O/L/s (37%) for Xrs_5_, and ±4.8 cmH_2_O/L (72%) for AX.^61^ Interestingly, the between-day repeatability measured in 31 healthy adults is very similar, being about ±1 cmH_2_O/L/s (30%) for Rrs_5_ and about ±0.5 cmH_2_O/L/s (54%) for Xrs_5_.^65^ In that study, the equivalent coefficient of repeatability but expressed as *z* scores was about ±1.0 for Rrs_5_ and ±0.5 for Xrs_5_.

BDR is a significant physiologic characteristic in asthma, albeit nonspecific. Spirometric BDR is associated with the development of fixed airflow obstruction,^66^ exacerbation risk,^67^ poor asthma symptom control,^68^ poor response to treatment in pediatric patients,^69^ and asthma death.^70^ The upper limit of BDR for oscillatory impedance in healthy subjects has been well documented,^61^^,^^63^^,^^71^ being about −1.4 cmH_2_0/L/s for Rrs_5_ and +0.6 cmH_2_O/L/s for Xrs_5_. These correspond to −1.4 *z* scores and +1.4 *z* scores, respectively.^63^ For AX, it was −4.0 cmH_2_O/L; the limits for Rrs_5_ − Rrs_20_ were unknown.

There has been only a single recent article that addresses the question of minimal clinically important difference (MCID).^72^ Changes in Rrs_5_ − Rrs_20_ and AX were referenced to asthma symptom scores in which a decrease of greater than or equal to 0.6 SD of symptom scores with treatment was considered to be clinically important. The MCIDs for Rrs_5_ − Rrs_20_ and AX were −0.6 cmH_2_O/L/s and −6.6 cmH_2_O/L, respectively. To put this into context, for AX, the MCID is similar to the lower limit of BDR. The limits for BDR for Rrs_5_ − Rrs_20_ are unknown, but typical limits are 0.6 to 0.7 cmH_2_O/L/s (or about 0.7 SD),^73^, ^74^, ^75^ that is, similar to its MCID. Similar studies on MCID for Rrs_5_, Xrs_5_, and intrabreath parameters are very much needed.

The magnitude of oscillatory impedance responses in relation to the variability of measurement (ie, signal to noise) is greater than for spirometry.^74^^,^^76^ However, a study of 33 subjects with asthma screened for clinical trials^76^ and another study of 122 subjects with asthma^63^ indicate that spirometry and oscillometry measurements produce similar proportions of positive BDR. Again, however, there is discordance between oscillometry and spirometry identifying different individuals with positive BDR. In another study of 52 patients attending a severe asthma service, discordant BDR between spirometry and oscillometry was again demonstrated.^77^ Interestingly, Xrs_5_ and AX identified positive BDR in 40% and 52%, respectively, compared with FEV_1_, which identified 27% having positive BDR. However, Xrs_5_ and AX BDR correlated with asthma control, whereas Rrs_5_ and spirometry BDR did not.^77^

Spirometry as well as Rrs and Xrs together provide a global assessment of airway function impairment and BDR. Spirometry reflected mostly proximal airway function, whereas oscillatory impedance reflected peripheral airway function also. As for all lung function measurements, it may inform disease activity, risk, and hence management decisions. For example, a persistently low Xrs with or without a persistently large BDR may suggest suboptimal control of bronchoconstriction, which may be deemed to be clinically significant, dependent on their clinical picture and progress. Impedance may also change over time and can be interpreted in the same way as spirometry, in that improvements may suggest favorable responses to treatment, whereas deterioration may suggest worsening. Table I summarizes the repeatability, lower and upper limits of BDR, and typical bronchodilator changes in asthma. On the basis of these figures, approximations for MCID for Rrs might be greater than 1 cmH_2_O/L/s and greater than 1.5 *z* scores, and for Xrs might be greater than 1 cmH_2_O/L/s and greater than 1.5 *z* scores. However, it is clear that future studies on relationships with other intermediate to long-term outcomes, such as loss of FEV_1_, ability to downtitrate, or day-to-day variability, would also be useful to inform MCID and hence clinical application.

**Table I:** Repeatability, suggested thresholds for bronchodilator responsiveness, and typical bronchodilator responses in asthma

	Between-day repeatability	BDR∗ thresholds (LLN or ULN)	“Typical” BDR in asthma†
Rrs_5_	1 cmH_2_O/L/s 30% 0.96 *z* scores	−1.4 cmH_2_O/L/s 30% −1.4 *z* scores	−0.6 to −1.0 cmH_2_O/L/s 10%-20% —
Xrs_5_	0.5 cmH_2_O/L/s 54% 0.47 *z* scores	0.6 cmH_2_O/L/s 45% 1.4 *z* scores	1-1.2 cmH_2_O/L/s 30%-50% —
AX	4.8 cmH_2_O/L — —	−4 cmH_2_O/L — —	−10 to −20 cmH_2_O/L 25%-50% —

Values are given as absolute values, percentage of mean or baseline, and *z* scores.

*LLN*, Lower limit of normal; *ULN*, upper limit of normal.

∗ Limits of normal response.^61^^,^^63^^,^^71^

† “Typical” BDR in asthma is the approximate magnitude of change observed in published data.^32^^,^^75^, ^76^, ^77^

#### Ventilation imaging

Peripheral airways disease is also measured by ventilation imaging, with many studies demonstrating correlations between reduced or nonventilation and oscillometry or MBNW.^39^^,^^46^^,^^48^^,^^58^^,^^78^, ^79^, ^80^, ^81^ Functional imaging measures of peripheral airways could become part of routine assessment of patients. Chest x-rays and CT scans are often used, more to exclude other diseases (eg, bronchiectasis or fibrosis) rather than to provide information specific to airways disease. Standardization of techniques, defining ventilation parameters from a normal population, and having well-developed and practical image analysis will allow adoption of probably SPECT and CT imaging as the most accessible and practical modalities.

#### Aerosol delivery

Principles of aerosol delivery to airways suggest that particles of the order of 1 to 3 μm deposit to the lung periphery, and this is supported by human experimental evidence.^82^^,^^83^ Furthermore, optimal inhalation rate and breath hold also have an impact on total deposition and penetration to the periphery. Treating severe asthma requires optimization of inhaled therapy, and therefore getting high ICS doses reaching the lungs and particularly to the lung periphery, at least for a defined trial period to determine true ICS resistance. To achieve this, clinicians should consider the use of aerosols with high fine-particle fractions (high fractions <5 μm in diameter) or extrafine formulations (at least half the dose having a diameter ≤2.1 μm), either solely or as add-on,^84^^,^^85^ and combined with optimal inhaler technique. Optimal inhalation flow rate is about 30 L/min,^86^ which may feel surprisingly slow to many patients, and about 5- to 10-second postinhalation breath hold. Holding chambers may be very effective and useful in most and should be assessed on an individual basis.

### Conclusions

Peripheral airways disease is fundamental to asthma because of the known anatomical and cellular pathology, pathophysiology from complex airway measurements and imaging, and the large body of evidence showing consistent clinical associations. Peripheral airways disease is probably most relevant in ICS-resistant (severe) asthma, and knowledge of targeting ICS/LABA treatment to the peripheral airway compartment can guide treatment strategies. A much greater understanding of peripheral airways disease is required, however, in relation to remodeling and type 2 low inflammation, because drug treatments other than ICS are desperately needed. Perhaps more systemic treatments will be used in future to treat severe asthma because they can access the peripheral airway compartment, bypassing the challenges of traversing many generations of airways to reach their targets. Current technologies including nitrogen washout, respiratory oscillometry, and imaging are undergoing rapid development to make them more practical as ways of measuring peripheral airway function, which will be useful for clinicians and patients to manage problematic asthma.

**Key messages**

• Peripheral airways disease in asthma is characterized by patchy airway narrowing and airway closure, which leads to ventilation heterogeneity.
• Ventilation heterogeneity due to peripheral airway dysfunction can be measured in clinical practice using respiratory oscillometry and multiple breath inert gas washout tests, whereas modern imaging techniques are an emerging technology.
• Peripheral airway tests complement spirometry but have a stronger base of clinical associations, which allows its interpretation within the clinical context of individual patients, for example, in assessing response to treatment in relation to exacerbation risk (MBNW) and lung function (oscillometry).
• Knowing the role of peripheral airways disease in those who have difficult-to-treat asthma, spirometric impairment, and who are older provides a rationale to consider aerosol targeting of the peripheral airway compartment.
• Measurement of peripheral airway function will become part of routine management and will be essential in developing new medications in asthma.

## Disclosure statement

Chiesi Australia supported the authors with the resources of a medical writer (George Krassas, Scius Healthcare Solutions), who has assisted with coordinating meetings of the author group, minuting decisions, and has assisted with the final formatting the manuscript for submission. There was no involvement in drafting or editing the manuscript which was done solely by the authors.

Disclosure of potential conflict of interest: G. G. King is a nonexecutive board member of Cyclomedica Australia Ltd and has received honoraria for consultative services to AstraZeneca, Boehringer Ingelheim, Chiesi, GSK, and Sanofi.  K. Nilsen is a full time employee of 4DMedical. B. R. Thompson is on the Medical Advisory Board of NDD, Chiesi, and 4DMedical. The rest of the authors declare that they have no relevant conflicts of interest.
